# Response to Maarten C. Eisma and Lonneke I. M. Lenferink

**DOI:** 10.1080/20008198.2018.1536288

**Published:** 2018-10-24

**Authors:** Clare Killikelly, Andreas Maercker

**Affiliations:** Department of Psychology, Division Psychopathology and Clinical Intervention, University of Zurich, Zürich, Switzerland

The introduction of prolonged grief disorder (PGD) as a new disorder in the ICD-11 was greatly debated. Several researchers thought that it might pathologize a normal human response to loss (Wakefield, 2012), while others asserted the importance of correctly diagnosing and treating a group of people who were in great distress (Prigerson & Maciejewski, 2017; Shear, Muldberg, & Periyakoil, 2017). Ultimately, this new diagnosis was included in the ICD-11 with the aim to improve clinical practice and guide treatment planning but also to ignite high quality research into the nature of the disorder, the validity of diagnostic guidelines and the development of evidence based treatment options (Maercker et al., 2013). We are pleased to see that this important research is already underway.

In their letter, Eisma and Lenferink (2018) describe two main concerns with our recent paper ‘Prolonged grief disorder for ICD-11: the primacy of clinical utility and international applicability.’ They disagree that the ICD-11 PGD definition (a) offers valid diagnostic guidelines for the inclusion of PGD in the ICD-11 and (b) provides the same valid symptom structure as precursor definitions. We would like to respond with two main points.

Firstly, we acknowledge and agree that these above concerns are important empirical research questions. At the time of the publication of the paper there were no direct empirical examinations of the new ICD-11 PGD guidelines, only post-hoc studies from previous or even reconstructed datasets. Throughout the paper we consistently encourage further examination and additional research to validate the new ICD-11 PGD guidelines, for example we outline that further research is needed to directly compare the PGD-2009 criteria with the ICD-11 criteria using network analysis. Additionally, we would argue that the precursor criteria PGD-2009 have previously been validated and, although different, most closely represent the ICD-11 PGD guidelines. For evidence of the comparable structure of ICD-11 PGD and PGD-2009 please we refer to the formative work from Maciejewski, Maercker, Boelen and Prigerson (2016). Admittedly, the diagnostic approach chosen in this 2016 study is just one option amongst other potential algorithms. The spirit of the WHO-developed diagnostic definitions is not to provide exact diagnostic algorithms but rather to leave the search for operational criteria for subsequent research around the world (such as new research from Mauro et al. (2018) and Boelen, Lenferink, Nickerson, and Smid (2018) which directly assess the validity of the ICD-11 definition and brings into question the specificity of the new ICD-11 PGD criteria).

Secondly, we re-assert the main aim of our paper. There is a fundamental shift in the remit of the ICD-11. Instead of further validation, specification and itemization of diagnostic criteria, the focus is on developing clinically useful diagnostic guidelines (First, Reed, Hyman, & Saxena, 2015). A potentially overlooked prerequisite to clinical utility is brevity of diagnostic features. The current form of the PGD definition in ICD-11 is an example of a brief definition that supports clinical utility. As noted by Lenferink and Eisma (2018), a higher symptom count in the diagnostic criteria is not always helpful; persistent complex bereavement disorder, as a disorder requiring further study in the DSM-5, can be diagnosed 37,650 ways whereas PGD only 48. The development of the briefer ICD-11 PGD guidelines may truly improve the specificity of the diagnosis by providing less complex disorder descriptions and by relying on clinical judgement instead of diagnostic algorithms.

This raises an important epistemological question: should one type of research be prioritized when considering the new ICD-11 PGD criteria? Research that follows the traditional methods of psychometric validation has an important place in the further development and refinement of the diagnostic items, however research that reflects the voices of the patients and clinicians should also be highlighted (i.e. the request for few disorder categories with flexible diagnostic guidance to allow for clinical judgement) (Evans et al., 2013; Reed, Mendonça Correia, Esparza, Saxena, & Maj, 2011). We hope that future research may confirm that diagnostic guidelines can be both psychometrically valid and useful in the clinical setting.
